# Detecting and Monitoring Porcine Hemagglutinating Encephalomyelitis Virus, an Underresearched Betacoronavirus

**DOI:** 10.1128/mSphere.00199-20

**Published:** 2020-05-06

**Authors:** Juan Carlos Mora-Díaz, Ronaldo Magtoto, Elizabeth Houston, David Baum, José Antonio Carrillo-Ávila, Gun Temeeyasen, Jeff Zimmerman, Pablo Piñeyro, Luis Giménez-Lirola

**Affiliations:** a Department of Veterinary Diagnostic and Production Animal Medicine, College of Veterinary Medicine, Iowa State University, Ames, Iowa, USA; b Andalusian Public Health System Biobank, Granada, Spain; University of Maryland School of Medicine

**Keywords:** coronavirus, ELISA, encephalomyelitis, porcine hemagglutinating encephalomyelitis virus, seroprevalence, vomiting and wasting disease, porcine encephalomyelitis virus

## Abstract

Members of family *Coronaviridae* cause a variety of diseases in birds and mammals. Porcine hemagglutinating encephalomyelitis virus (PHEV), a lesser-researched coronavirus, can infect naive pigs of any age, but clinical disease is observed in pigs ≤4 weeks of age. No commercial PHEV vaccines are available, and neonatal protection from PHEV-associated disease is presumably dependent on lactogenic immunity. Although subclinical PHEV infections are thought to be common, PHEV ecology in commercial swine herds is unknown. To begin to address this gap in knowledge, a serum IgG antibody enzyme-linked immunosorbent assay (ELISA) based on the S1 protein was developed and evaluated on known-status samples and then used to estimate PHEV seroprevalence in U.S. sow herds. Assessment of the diagnostic performance of the PHEV S1 ELISA using serum samples (*n* = 924) collected from 7-week-old pigs (*n* = 84; 12 pigs per group) inoculated with PHEV, porcine epidemic diarrhea virus, transmissible gastroenteritis virus, porcine respiratory coronavirus, or porcine deltacoronavirus showed that a sample-to-positive cutoff value of ≥0.6 was both sensitive and specific, i.e., all PHEV-inoculated pigs were seropositive from days postinoculation 10 to 42, and no cross-reactivity was observed in samples from other groups. The PHEV S1 ELISA was then used to estimate PHEV seroprevalence in U.S. sow herds (19 states) using 2,756 serum samples from breeding females (>28 weeks old) on commercial farms (*n* = 104) with no history of PHEV-associated disease. The overall seroprevalence was 53.35% (confidence interval [CI], ±1.86%) and herd seroprevalence was 96.15% (CI, ±3.70%).

**IMPORTANCE** There is a paucity of information concerning the ecology of porcine hemagglutinating encephalomyelitis virus (PHEV) in commercial swine herds. This study provided evidence that PHEV infection is endemic and highly prevalent in U.S. swine herds. These results raised questions for future studies regarding the impact of endemic PHEV on swine health and the mechanisms by which this virus circulates in endemically infected populations. Regardless, the availability of the validated PHEV S1 enzyme-linked immunosorbent assay (ELISA) provides the means for swine producers to detect and monitor PHEV infections, confirm prior exposure to the virus, and to evaluate the immune status of breeding herds.

## Abstract (precis)

There is a paucity of information concerning the ecology of porcine hemagglutinating encephalomyelitis virus (PHEV) in commercial swine herds. This study provided evidence that PHEV infection is endemic and highly prevalent in U.S. swine herds. These results raised questions for future studies regarding the impact of endemic PHEV on swine health and the mechanisms by which this virus circulates in endemically infected populations. Regardless, the availability of the validated PHEV S1 enzyme-linked immunosorbent assay (ELISA) provides the means for swine producers to detect and monitor PHEV infections, confirm prior exposure to the virus, and to evaluate the immune status of breeding herds.

## INTRODUCTION

Coronaviruses (order *Nidovirales*, suborder *Cornidovirineae*, family *Coronaviridae*, subfamily *Orthocoronavirinae*) are enveloped, single-strand, positive-sense RNA viruses capable of infecting a wide variety of bird and mammal species (1, 2). These viruses often produce subclinical to mild respiratory or enteric infections. However, there are some recently re/emerged coronaviruses that cause severe clinical diseases in pigs, e.g., the *Alphacoronavirus* porcine epidemic diarrhea virus (PEDV) (3) and porcine deltacoronavirus (PDCoV) (4), and in humans, e.g., the betacoronaviruses Middle East respiratory syndrome (MERS) virus (5), severe acute respiratory syndrome (SARS) virus (6), and most recently, SARS-CoV-2 (7).

Overall, human and animal coronaviruses exhibit a marked propensity to recombine, mutate, and infect multiple species and cell types (1, 8). This propensity for interspecies transmission justifies close examination of members of this family. Porcine hemagglutinating encephalomyelitis virus (PHEV), the focus of this study, is a swine coronavirus in the genus *Betacoronavirus* and related to other *Betacoronavirus 1* species, including bovine coronavirus (BCoV), human coronavirus OC43 (HCoV-OC43), equine coronavirus (ECoV), and canine respiratory coronavirus (CrCoV). PHEV-associated disease was first described in Canada in 1957 in nursery pigs exhibiting vomiting, anorexia, constipation, and severe progressive emaciation (9). PHEV can produce vomiting and wasting disease (VWD) and/or encephalomyelitis, with mortality rates reaching 100% in neonatal pigs. Both clinical forms may occur simultaneously within a herd during an outbreak (10). Clinical PHEV was subsequently reported in Belgium (11), China (12–14), Argentina (15), South Korea (16), and the United States (17). More recently (2015), PHEV was associated with a case of influenza-like respiratory disease in 2015 in show pigs at a county fair in Michigan (18).

Although PHEV was isolated in 1962 (19), a search of PubMed for “porcine hemagglutinating encephalomyelitis virus” produced only 40 refereed publications since 1981, the majority of which address basic research questions. In contrast, information regarding the epidemiology or ecology of PHEV in contemporary farms is nearly entirely absent; even the seroprevalence of PHEV in most countries, including the United States, is unknown. To begin to address this shortfall, a serum IgG enzyme-linked immunosorbent assay (ELISA) based on the amino terminal portion (S1) of the PHEV spike protein was developed and evaluated under experimental conditions. Thereafter, the PHEV S1 ELISA was used to estimate the seroprevalence of PHEV in sow herds in the United States.

## RESULTS

### Diagnostic performance of PHEV S1 indirect ELISA.

**FIG 1:**
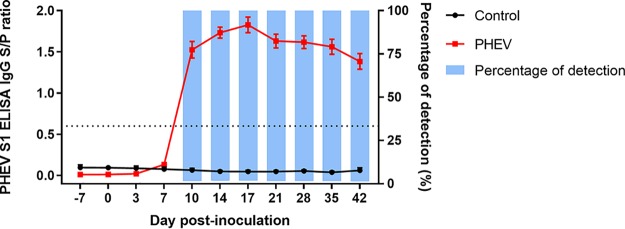
PHEV S1 ELISA sample-to-positive (S/P) ratios of serum IgG responses. Each line represents the dynamic of PHEV antibodies in PHEV- and control-inoculated groups. Each time point is represented by the S/P mean and standard errors. Colored bars represent the percentages of positive samples over time in pigs experimentally inoculated with PHEV (Mengeling strain; *n* = 12) or mock inoculated with culture medium (control group; *n* = 12). Samples with an S/P ratio of >0.6 (dashed line) were considered seropositive to PHEV.

**FIG 2:**
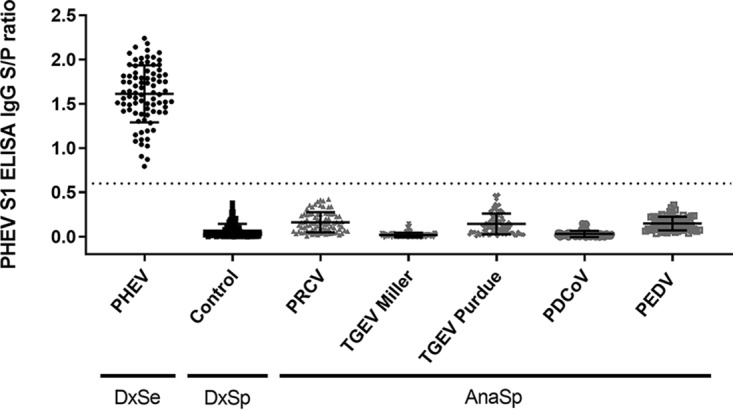
Distribution of cumulative PHEV S1 IgG ELISA sample-to-positive (S/P) ratios from experimental serum samples collected at −7, 0, 3, 10, 14, 17, 21, 28, 35, and 42 dpi from pigs inoculated with PHEV (Mengeling strain; *n* = 12) to evaluate the diagnostic sensitivity (DxSe), mock inoculated with culture medium (*n* = 12) to evaluate the diagnostic specificity (DxSp), and inoculated with PEDV (USA/IN/2013/19338E; *n* = 12), TGEV Miller (ATCC VR-1740; *n* = 12), TGEV Purdue (ATCC VR-763; *n* = 12), PRCV (ATCC VR-2384; *n* = 12), or PDCoV (USA/IL/2014; *n* = 12) to evaluate the analytical specificity (AnaSp). Samples above the S/P 0.6 cutoff (dashed line) were considered positive.

A cutoff sample-to-positive (S/P) ratio of ≥0.6 was selected based on a receiver operating characteristic (ROC) analysis performed on ELISA results from known-status samples. Based on this cutoff, a PHEV-specific IgG response was observed in all PHEV-inoculated pigs (12/12) from days postinoculation (dpi) 10 through 42, thereby providing a diagnostic sensitivity of 100% (Fig. 1). No PHEV S1 ELISA-positive responses were observed with samples from pigs in the negative group (Fig. 1) or samples from pigs inoculated with other porcine coronaviruses (i.e., PEDV, transmissible gastroenteritis virus [TGEV] Purdue, TGEV Miller, porcine respiratory coronavirus [PRCV], and PDCoV), providing 100% diagnostic and analytical specificity (Fig. 2).

### Seroprevalence of PHEV in sow herds in the United States.

**FIG 3:**
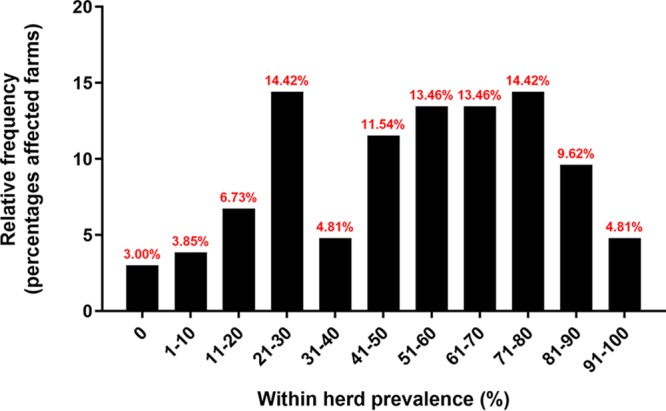
Within-herd seroprevalence distribution in the United States. Bars represent the frequency of farms (*n* = 104) with different levels of within-herd seroprevalence. Only the results of herds with at least two positive sows were displayed.

**FIG 4:**
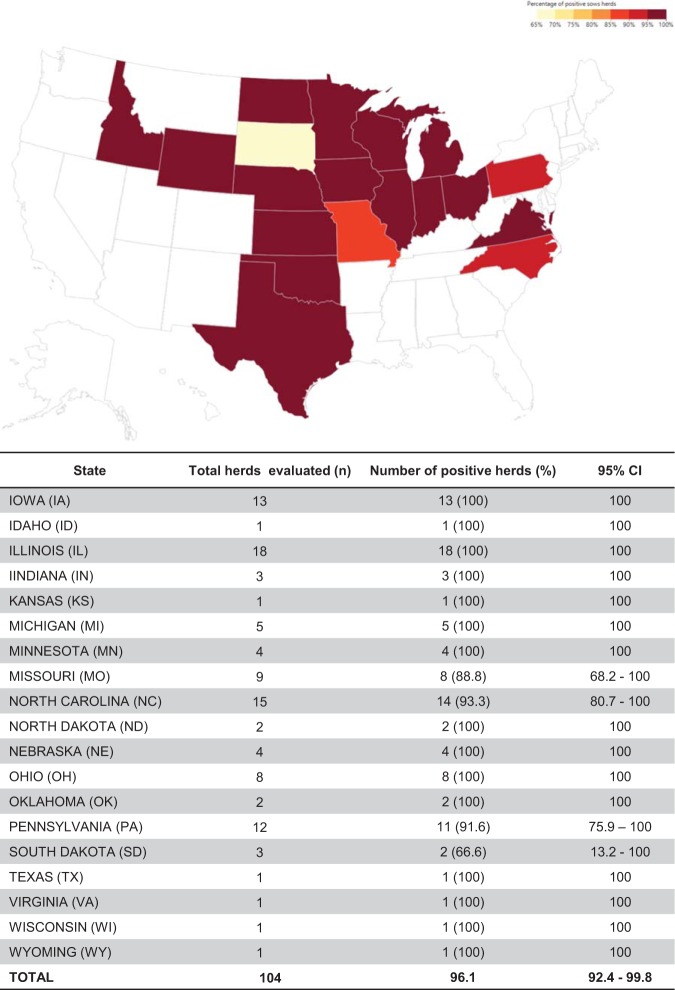
Geographic distribution of PHEV seropositive farms (herd prevalence by state) in the United States. The map indicates the percentages of PHEV-seropositive herds by U.S. state. The table indicates the total numbers evaluted and percentages of positive farms by state and 95% confidence intervals (CIs).

**FIG 5:**
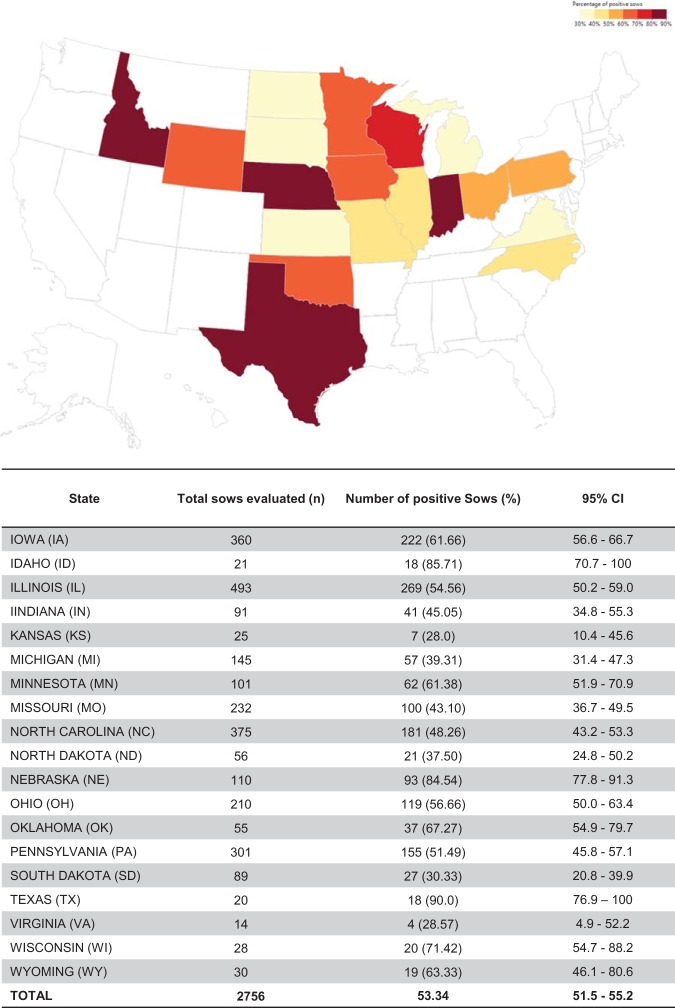
Geographic distribution of PHEV seropositive sows (overal invididual prevalence by state) in the United States. The map indicates the percentages of PHEV-seropositive sows detected by state. The table indicates the total numbers evaluated and percentages of positive sows by state with the 95% confidence intervals (CIs).

The overall seroprevalence at individual (*n* = 2,756) and herd (*n* = 104) levels was 53.35% (confidence interval [CI], ± 1.86%) and 96.15% (CI, ±3.70%), respectively. Among positive farms, within-herd prevalence ranged from 1% to 50%, 51% to 70%, and 71% to 100% in 41.3%, 26.9%, and 28.8% of herds, respectively (Fig. 3). The prevalence of positive sows and herds by state is shown in Fig. 4 and 5. Although the field survey was not stratified by herd size or number of herds within states, all 19 states represented in the study had at least one seropositive farm (Fig. 5).

## DISCUSSION

Recent decades have seen an increase in the occurrence of infectious diseases of swine, and porcine coronaviruses are part of this trend. TGEV, the first porcine coronavirus to be identified, was described in 1946 (20). This was followed by PHEV in 1961 (19), PEDV in 1976 (21), PRCV (a spike gene-deletion TGEV mutant) in 1986 (22), PDCoV in 2012 (4), and swine acute diarrhea syndrome coronavirus in 2017 (23). The complexity of the situation further increased with the appearance of highly virulent PEDV in China (2010) and elsewhere (3, 24) and, more recently, the recognition of recombinant porcine coronaviruses, e.g., the TGEV-PEDV recombinants (SeCoV) identified in Italy (25), Germany (26), and Slovakia (27).

Among the porcine coronaviruses, PHEV is the most poorly documented, even the frequency of PHEV infections in the field is unknown. Antibody testing is an accurate, efficient, and cost-effective method to collect population data, but laboratory throughput and antibody cross-reactivity between porcine coronavirus species can be problematic. Previous studies reported PHEV antibody detection by hemagglutination-based assays, virus neutralization (VN) tests, ELISA, and immunochromatographic-based tests (28–31), but their diagnostic performance under experimental or field conditions have not been assessed. Like all coronavirus, PHEV possesses four structural proteins: spike glycoprotein (S), nucleocapsid protein (N), small membrane protein (E), and transmembrane glycoprotein (M). In addition, similar to other *Betacoronavirus 1* species, PHEV possesses a layer of envelope-associated glycoproteins (hemagglutinin-esterase) (2). With the exception of the intractable and well-documented cross-reactivity between TGEV and PRCV (32), serological differentiation among porcine coronaviruses can only been achieved by utilizing the S1 domain as the antigen in species-specific antibody tests (33). Therefore, in the present study, the PHEV S1 protein was used as antigenic target instead of more conserved proteins (S2 domain, N, and M), because it contains major antigenic determinants distinct from those of other coronaviruses. The PHEV recombinant S1 protein was expressed under native (soluble) conditions using a eukaryotic (mammalian) expression system to preserve essential conformation and/or glycosylation-dependent epitopes and then used in an indirect ELISA for IgG antibody detection in serum samples.

The lack of diagnostic specimens from pigs of precisely known infection status is the most common obstacle to the accurate assessment of the diagnostic performance of any diagnostic tool, regardless of platform. In this study, the diagnostic performance of the PHEV S1 ELISA was assessed using samples of precisely known infection status. Diagnostic specificity was assessed using porcine coronavirus-negative pigs, the time to detection and diagnostic sensitivity were evaluated using samples from pigs experimentally inoculated with PHEV, and the analytical specificity of the test was evaluated using serum samples from pigs inoculated with other porcine coronaviruses (TGEV, PRCV, PEDV, and PDCoV). Results from experimental samples showed that a cutoff S/P ratio of≥ 0.6 on the PHEV S1 ELISA detected seroconversion in all PHEV-inoculated animals at ≥10 dpi and provided 100% diagnostic and analytical specificities, i.e., no cross-reactivity with other porcine coronaviruses was observed.

Prior to this study, the seroprevalence of PHEV in the U.S. pig population had not been investigated. Seroprevalence represents cumulative exposure to infection, and given the long duration of specific antibodies, it is less affected by seasonality or short-term fluctuations in transmissions than other parameters. This study confirms that PHEV is endemic in U.S. swine herds, with PHEV seroprevalence slightly higher in U.S. states with a higher pig density, i.e., Iowa, North Carolina, and Minnesota. The high seroprevalence of PHEV in sow farms juxtaposed with the general lack of reported clinical cases of VWD suggests a scenario in which clinical signs are either unrecognized and/or piglets are fully or partially protected against PHEV through lactogenic immunity. Further research is needed to better understand the ecology of PHEV in commercial swine herds and the role of lactogenic immunity in the protection of piglets against PHEV. Regardless, given their proven ability to mutate, recombine, and colonize new niches, maintaining a level awareness concerning the potential impact of porcine coronaviruses on animal health, public health, and the food supply would be prudent.

## MATERIALS AND METHODS

### Experimental design.

With the approval of the Iowa State University (ISU) Institutional Animal Care and Use Committee, 7-week-old piglets (*n* = 84) naive for porcine coronavirus infections were allocated to 7 inoculation groups: negative (mock) control, PHEV, PEDV, porcine respiratory coronavirus (PRCV), transmissible gastroenteritis virus (TGEV) Miller strain, TGEV Purdue strain, and PDCoV. Serum samples collected at −7, 0, 3, 7, 10, 14, 17, 21, 28, 35, and 42 days postinoculation (dpi) were used to optimize the PHEV S1 ELISA and evaluate antibody cross-reactivity among porcine coronaviruses. Thereafter, 2,756 serum samples submitted to the ISU Veterinary Diagnostic Laboratory for routine testing from 104 farms in 19 states were used to assess the seroprevalence in sow herds in the United States.

### Generation of PHEV recombinant S1 protein.

The coding region of the receptor-binding (S1) domain sequence derived from PHEV strain VW572 (GenBank DQ011855.1) (34) was synthetically produced (Shanghai Generay Biotech Co., Ltd., China) with the addition of a 5′ terminal eukaryotic native signal (MFFILLISLPSAFAVIG), a 3′ terminal Tobacco etch virus (TEV) cysteine protease site (ENLYFQS), and the Fc portion of human IgG1 (GenBank JX292764.2). The gene was amplified using the forward primers F1 (5′-AAACGGATCTCTAGCGAATTCGCCGCCACCATGTTCTTCATTCTGCTCATC-3′) and F2 (5′-GAGAACCTGTACTTCCAGAGCGACAAAACTCACACATGCC-3′) and reverse primers R1 (5′-GCTCTGGAAGTACAGGTTCTCGCGCCTACTCCGCAGGGCGG-3′) and R2 (5′-CGAGCGGCCGCTAGCAAGCTTTCATTTACCCGGAGACAGGG-3′). The amplicon was cloned into a eukaryotic expression vector (pNPM5; Novoprotein, Short Hills, NJ, USA), and the plasmid was transiently transfected into human embryonic kidney (HEK) 293 cells (1 × 10^6^ cells/ml) (Invitrogen of Thermo Fisher Scientific, Grand Island, NY, USA) using polyethylenimine (PEI) (Thermo Fisher Scientific). Transfected HEK293 cells were grown in serum-free FreeStyle 293 Expression medium (Gibco of Life Technologies, Carlsbad, CA, USA) at 37°C, 5% CO_2_, and shaking at 120 rpm. Five days after transfection, the culture supernatant was harvested by centrifugation at 3,500 × *g* for 20 min and filtered using 0.45-μm filters (Corning, Corning, NY, USA). The soluble expression of Fc-fused S1 protein (1,005 amino acids [aa], 112.3 kDa) was confirmed by dodecyl sulfate-polyacrylamide (12%) gel electrophoresis (SDS-PAGE). The Fc tag was enzymatically cleaved by incubation with TEV (20 IU/mg sample) for 3 h at 25°C under endotoxin control, to rule out potential contaminations with bacterial endotoxins. PHEV S1 was purified using protein A affinity chromatography (GE Healthcare, Pittsburgh, PA, USA) and nickel (Ni)-chelating Sepharose Fast Flow (SFF) affinity chromatography (GE Healthcare), according to the manufacturer’s instructions. Purified PHEV S1 (754 aa) protein was dialyzed against phosphate-buffered saline (PBS; pH 8) and analyzed by SDS-PAGE and Western blotting.

### PHEV S1 ELISA.

PHEV S1 protein was coated (100 μl per well; 0.94 μg/ml in PBS [pH 7.4]) on 96-well plates (Immuno Breakables Modules; Thermo Fisher Scientific) and plates were incubated at 4°C for 16 h. After incubation, plate wells were washed five times with 300 μl PBS (pH 7.4) containing 0.1% Tween 20 (PBST), blocked with a 1% (wt/vol) bovine serum albumin solution (Jackson ImmunoResearch Inc., West Grove, PA, USA), incubated at 25°C for 2 h, dried at 37°C for 3 h, sealed, and stored at 4°C until use. Samples and positive and negative controls (100 μl per well; controls tested in duplicates) were diluted at 1:100 and incubated at 37°C for 1 h. Plates were then washed five times with PBST as described above and then incubated with 100 μl of a 1:35,000 dilution of peroxidase-conjugated goat anti-pig IgG (Fc) antibody (Bethyl Laboratories, Inc., Montgomery, TX, USA) at 37°C for 1 h. The reaction was revealed by adding 100 μl of tetramethylbenzidine-hydrogen peroxide (TMB) substrate solution (SurModics IVD, Inc., Eden Prairie, MN, USA) per well; the reaction was incubated at room temperature for 5 min and stopped by adding 100 μl of stop solution per well (SurModics IVD, Inc.). The optical density at 450 nm (OD_450_) was measured using an ELISA plate reader (BioTek Instruments, Inc., Winooski, VT, USA) operated with commercial software (SoftPro 7; Molecular Devices, San Jose, CA, USA). Antibody responses were expressed as sample-to-positive (S/P) ratios: S/P ratio = (sample OD_450_ − negative-control mean OD_450_)/(positive-control mean OD_450_ − negative-control mean OD_450_).

### Serum samples of known PHEV status.

Eighty-four 7-week-old pigs were selected from a wean-to-finish farm with no history of porcine coronavirus (PorCoV) infection. The pigs were prescreened negative for specific PorCoVs, i.e., PHEV, PEDV, TGEV, PRCV, and PDCoV, as described elsewhere (33). Animals were randomly placed into 7 treatment groups. Pigs within each group (*n* = 12) were distributed in six pens, i.e., two pigs per pen. One group was inoculated oronasally with 0.5 ml (1:128 hemagglutinin [HA] titer) PHEV Mengling strain (National Veterinary Services Laboratories [NVSL], United States Department of Agriculture [USDA], Ames, IA, USA) and used to evaluate the diagnostic sensitivity of the PHEV S1 ELISA described herein. A negative-control group was mock inoculated oronasally with Eagle’s minimum essential medium (EMEM) (ATCC, Manassas, VA, USA) and used to evaluate the diagnostic specificity of the test. The analytical specificity (cross-reactivity) of the test was evaluated on pigs exposed to PEDV (USA/IN/2013/19338E), TGEV Purdue (ATCC VR-763), TGEV Miller (ATCC VR-1740), PRCV (ATCC VR-2384), or PDCoV (USA/IL/2014) under experimental conditions as described elsewhere (33).

A total of 924 samples were collected from individual pigs across all treatment groups at −7, 0, 3, 7, 10, 14, 17, 21, 28, 35, and 42 days postinoculation (dpi). The pigs were evaluated twice daily for clinical signs throughout the study. At the end of the observation period, pigs were euthanized by the use of a penetrating captive bolt (Accles and Shelvoke, Ltd., Sutton Coldfield, United Kingdom).

### Field samples.

Serum samples were selected from regular submissions for porcine reproductive and respiratory syndrome virus testing at the ISU Veterinary Diagnostic Laboratory from June to December 2016. A total of 2,756 samples from gilts (>26 weeks of age) and sows were obtained from 104 farms with no history of PHEV-compatible clinical disease located in 19 U.S. states, i.e., an average of 24 serum samples per farm. Samples were stored at −80°C until use.

### Data analysis.

The PHEV S1 IgG ELISA cutoff value was determined by receiver operator characteristic (ROC) curve analysis (SAS version 9.4; SAS Institute, Inc., Cary, NC, USA). Serum samples (*n* = 72) collected at ≥10 dpi from the PHEV-inoculated group were considered antibody positive and used to evaluate the diagnostic sensitivity. The diagnostic specificity was evaluated using serum samples from PorCoV-negative pigs, i.e., all samples collected from the negative group and samples collected from all pigs across groups prior to inoculation (−7 and 0 dpi) among all treatment groups (*n* = 266). The analytical specificity of the test was evaluated using samples (*n* = 440) collected from animals inoculated with PEDV, TGEV Miller strain, TGEV Purdue strain, PRCV, and PDCoV from 7 to 42 dpi. The PHEV S1 ELISA S/P serum IgG responses obtained for each inoculation group were plotted using GraphPad Prism (GraphPad Software Inc., La Jolla, CA, USA).

For the analysis of field sample data, farms with ≥2 PHEV S1 ELISA-positive serum samples were considered positive for PHEV. The overall individual and herd U.S. seroprevalence as well as the geographic distribution of PHEV by state (individual and herd level) with a 95% conﬁdence intervals (CIs) were estimated for the total number of serum samples tested (*n* = 2,756) using the PHEV S1 ELISA. Statistical analyses and geographical plots were performed using JMP 13.1.0 (SAS Institute Inc., Cary, NC, USA). The within-herd prevalence frequency was estimated using GraphPad Prism.
